# Draft genome sequences of five *Bartonella henselae* strains from shelter cats in Osaka, Japan

**DOI:** 10.1128/mra.00904-25

**Published:** 2025-11-28

**Authors:** Kaoru Umeda, Yuji Hirai

**Affiliations:** 1 Department of Microbiology, Osaka Institute of Public Health 91397 , Osaka, Japan; Fluxus Inc., Sunnyvale, California, USA

**Keywords:** *Bartonella henselae*, zoonosis, shelter cats, draft genome sequences

## Abstract

We report the draft genome sequences of five *Bartonella henselae* strains detected in the blood of cats in Osaka, Japan. The average genome size was 1.85 Mbp, and the average guanine–cytosine content was 38%. All strains belonged to sequence type 1, the most common type among cat and human clinical isolates.

## ANNOUNCEMENT

*Bartonella henselae* is a facultative intracellular bacterium harbored by cats that can be transmitted to humans, causing cat scratch disease (1, 2).

Whole-genome information is important for analyzing *B. henselae* pathogenicity and transmissibility. However, isolating *B. henselae* from the blood of healthy cats is challenging due to its fastidious nature and slow growth (3).

In 2017–2019, blood samples were collected from cats at an Osaka animal shelter, Japan (34°37′12.7″N 135°27′56.2″ E). The samples were surplus from default blood tests conducted on housed animals and did not correspond to animal experiments. Animals were cared for in accordance with the Japanese laws. Samples were stored at −20°C until use. *B. henselae* was detected in 16 of 193 samples using a PCR-based method (4, 5). Of these, eight strains were isolated from eight cats after a 4-week culture of 200 µL blood samples on Trypticase soy agar (TSA) II 5% sheep blood agar (Becton Dickinson, US) at 35°C with 5% CO_2_. Taxonomic identity was confirmed using partial 16S rRNA sequencing (6). Briefly, DNA was isolated using the DNeasy Blood & Tissue Kit (Qiagen, Germany) according to the manufacturer’s instructions for gram-negative bacteria. Gene fragments were amplified using BAKllw/PC3mod primers (6) and sequenced by the Sanger sequencing method using the BigDye Terminator v3.1 Cycle Sequencing Kit and the ABI 3500 Genetic Analyzer (Applied Biosystems, USA). These were identical to reference sequences (*B. henselae* FDAARGOS_1482, NZ_CP082885). Five strains were selected based on differences in multiple bands in the *Sma-*I-digested pulsed-field gel electrophoresis.

For DNA isolation, stock strains were grown on TSA II 5% sheep blood agar M (Becton Dickinson) for 3 days at 35°C with 5% CO_2_ without subculturing. Genomic DNA was extracted from single colonies scraped from the agar using the Illustra Bacteria Genomic Prep Mini Spin Kit (Cytiva, Japan), according to the manufacturer’s instructions for gram-negative bacteria. DNA purity and concentration were calculated using NanoDrop (Thermo Scientific, USA) and Qubit (Invitrogen, USA), respectively.

The short-read library was prepared using the Nextera DNA Flex Library Prep Kit (Illumina, USA) and sequenced on iSeq 100 (Illumina) to produce paired-end 2×150 bp reads. Illumina sequence data were trimmed and *de novo* assembled with a 500 bp minimum contig size using CLC Genome Workbench ver.25.0 (Qiagen) using the default settings.

The sequences were quality-controlled using CLC Genomic Workbench ver.25.0 (Qiagen) to confirm coverage (minimum ×30). DFAST pipeline ver.1.6.0 (https://dfast.ddbj.nig.ac.jp/) (7) was used for species verification by calculating average nucleotide identity (ANI) value (%) to reference sequences (*B. henselae* Houston-I, GCA_002735245.1) and to check contamination and completeness by selecting the “Completeness check” option. Annotation was performed using DFAST ver.1.6.0 (8), and the sequence type (ST) for MLST was identified using the PubMLST database (https://pubmlst.org/) (9). Default parameters were used, except where otherwise noted.

Genomic information is presented in Table 1. Between 73 and 84 contigs per strain were constructed with a coverage of >77×. The average genome size was 1.85 Mbp with 1,522 coding sequences and 38% GC content, comparable to that of *B. henselae* strains in the literature. All strains belonged to ST1, the most common type among cat and human clinical isolates (10, 11).

**TABLE 1:** Genomic information of *B. henselae* isolates from cats in Japan

Strain ID	Genome size (bp)	Total read length (bp)	Genome coverage (×)	GC content (%)	Number of contigs	N_50_ (bp)	Completeness (%)	Number of coding sequences	ST
OIPH-60-1	1,838,349	141,715,021	77.1	37.9	73	82,710	99.95	1,514	1
OIPH-63-1	1,852,104	153,224,552	82.7	38.0	79	90,634	99.95	1,526	1
OIPH-76-1	1,848,234	120,637,181	65.3	38.0	84	90,634	99.95	1,517	1
OIPH-79-1	1,840,248	155,885,275	84.7	37.9	78	90,634	99.95	1,523	1
OIPH-80-1	1,852,095	172,184,648	88.2	38.0	77	90,685	99.95	1,529	1

## Data Availability

The whole-genome shotgun projects/sequence read archives have been deposited in DDBJ/ENA/GenBank under the accession numbers BAAHMW000000000/DRX667350 (OIPH-60-1), BAAHMX000000000/DRX667351 (OIPH-63-1), BAAHMY000000000/DRX667352 (OIPH-76-1), BAAHMZ000000000/DRX667353 (OIPH-79-1), and BAAHNA000000000/DRX667354 (OIPH-80-1). The versions described in this paper are the first versions.
